# Hemolytic Performance in Two Generations of the Sputnik Left Ventricular Assist Device: A Combined Numerical and Experimental Study

**DOI:** 10.3390/jfb13010007

**Published:** 2022-01-12

**Authors:** Alexandra N. Romanova, Alexander A. Pugovkin, Maxim V. Denisov, Ivan A. Ephimov, Dmitry V. Gusev, Marian Walter, Thomas Groth, Olga L. Bockeria, Tatyana G. Le, Anna S. Satyukova, Sergey V. Selishchev, Dmitry V. Telyshev

**Affiliations:** 1 Institute of Biomedical Systems, National Research University of Electronic Technology, 124498 Zelenograd, Moscow, Russia; romanova@bms.zone (A.N.R.); denisov@bms.zone (M.V.D.); ephimov@zitc-mt.ru (I.A.E.); dgusev@zitc-mt.ru (D.V.G.); sersel@miee.ru (S.V.S.); telyshev@bms.zone (D.V.T.); 2 Center for Digital Biodesign and Personalized Healthcare, I. M. Sechenov First Moscow State Medical University, 119991 Moscow, Russia; thomas.groth@pharmazie.uni-halle.de; 3 Medical Information Technology, Helmholtz Institute of Biomedical Engineering, RWTH Aachen University, 52074 Aachen, Germany; walter@hia.rwth-aachen.de; 4 Department Biomedical Materials, Institute of Pharmacy, Martin Luther University Halle-Wittenberg, 06120 Halle (Saale), Germany; 5 Bakulev Scientific Center for Cardiovascular Surgery, 121552 Moscow, Russia; olbockeria@bakulev.ru (O.L.B.); tgle@bakulev.ru (T.G.L.); assatyukova@bakulev.ru (A.S.S.)

**Keywords:** computational fluid dynamics, hemolysis, left ventricular assist device, mechanical circulatory support, shear stress

## Abstract

Background: Currently, left ventricular assist devices (LVADs) are a successful surgical treatment for patients with end-stage heart failure on the waiting list or with contraindicated heart transplantation. In Russia, Sputnik 1 LVAD was also successfully introduced into clinical practice as a bridge-to-transplant and a destination therapy device. Development of Sputnik 2 LVAD was aimed at miniaturization to reduce invasiveness, optimize hemocompatibility, and improve versatility for patients of various sizes. Methods: We compared hemolysis level in flow path of the Sputnik LVADs and investigated design aspects influencing other types of blood damage, using predictions of computational fluid dynamics (CFD) and experimental assessment. The investigated operating point was a flow rate of 5 L/min and a pressure head of 100 mm Hg at an impeller rotational speed of 9100 min^−1^. Results: Mean hemolysis indices predicted with CFD were 0.0090% in the Sputnik 1 and 0.0023% in the Sputnik 2. Averaged values of normalized index of hemolysis obtained experimentally for the Sputnik 1 and the Sputnik 2 were 0.011 ± 0.003 g/100 L and 0.004 ± 0.002 g/100 L, respectively. Conclusions: Obtained results indicate obvious improvements in hemocompatibility and sufficiently satisfy the determined miniaturization aim for the Sputnik 2 LVAD development.

## 1. Introduction

Heart failure (HF) is one of the most dangerous and widespread diseases in the world. For end-stage HF patients, therapeutic methods cannot ensure recovery, and heart transplantation becomes the only effective treatment. Unfortunately, number of patients requiring transplantation is considerably greater than number of donor organs available [1]. Since that, emergence of wearable artificial devices supporting or partially replacing human blood circulation has become an important step in the development of medicine, allowing patients suffering the end-stage heart failure to lead normal life outside the clinics. One of these devices is continuous-flow left ventricular assist device (LVAD), a complex system based on a rotary blood pump (RBP) of axial or centrifugal type [2,3]. LVAD supports pumping function of the left ventricle, the main pumping chamber of the heart. However, clinical benefits and cost-effectiveness of LVAD therapy remain limited [4,5]. If use of rotary blood pumps continues to grow in the future, it is necessary to improve expectancy and quality of patients’ life with reduced level of adverse events and therapy costs. As it is generally agreed that most adverse events are related to the device impact on the blood, further research is aimed at clarification of the causes and focused on development of less traumatic pumps.

For the development of LVADs, modern methods of computational fluid dynamics (CFD) are used along with conventional experimental assessment, allowing to investigate the blood flow field inside the device and to predict and evaluate possible blood damage [6,7,8,9,10,11,12,13,14,15,16]. Overall blood damage changes the coagulation cascade and may lead to thrombosis, embolism and decreased leukocyte functionality. The main problem for RBPs is determination of the level of different damage types inside the device including hemolysis, platelet activation, and degradation of von Willebrand factor (vWF) that attaches platelets to a damaged vessel. All mentioned types of blood damage are strongly dependent on shear stress in the blood flow field, especially in the wall region of flow path. On the one hand, low shear stress and stagnation of blood may affect platelet deposition and thrombosis. On the other hand, high shear stress may lead to hemolysis and platelet activation [8,10,11,12,14,15,16,17].

Impact on red blood cells (RBCs) leads to morphological and biochemical changes, decreased life expectancy or complete rupture of RBCs resulting in hemoglobin release into blood plasma. An increase in hemoglobin release can lead to a decrease in the content of oxygen and carbon dioxide, and can also cause kidney saturation, since free hemoglobin is toxic [18]. It has been found that a kidney can clear 14 g of free hemoglobin per day in a healthy person [19]. Researches on deformation and rupture of RBCs showed that shear stress and time of exposure to shear stress are the most important parameters evaluating blood damage in LVADs [20,21,22,23,24,25,26].

Since high rates of adverse events are linked to flow field within the pump, optimization of geometric parameters in the flow path is crucial. Generally, the main flow path elements in axial RBP are a flow straightener, a diffuser and a rotor represented by an impeller. All these elements have a certain number of blades of various geometric shapes. The basic operating principle of axial pumps is transfer of kinetic energy from the impeller blades to the fluid. When the fluid accelerated by the impeller runs on stationary blades of the diffuser, kinetic energy is converted into potential pressure energy, while the direction of flow is aligned along the central axis of flow path. In general, occurrence and amount of hemolysis are influenced by various changes in geometric parameters of flow path [27,28,29,30,31,32,33,34,35,36,37,38,39], for example, by changes in size of clearance gap between impeller blades and flow path housing. Smaller gaps induce increased values of shear stress, and larger gaps induce lesser values of shear stress, but increase the exposure time due to appearance of leakages, back flows and recirculation zones [27,28,29,38]. Additionally, optimization of profile and number of impeller and diffuser blades makes an increase of pump hydraulic efficiency possible, leading to a decrease in the value of impeller rotational speed at a design point, and therefore, to a decrease of impact on the blood [30,31,32,33]. Therefore, it is important to investigate parameters of a particular flow path determining shear stress magnitude and time of exposure to shear stress in different ranges.

In the present study, flow fields in the Sputnik LVADs of the first generation (Sputnik 1) and the second generation (Sputnik 2) were compared to investigate design aspects influencing the blood damage. Although Sputnik 1 LVAD was successfully introduced into clinical practice in Russia as a bridge-to-transplant and a destination therapy device [40], Sputnik 2 LVAD was developed to reduce power consumption and invasiveness and to improve versatility for patients of various sizes [41]. Study with mock circulatory loop revealed better hydraulic performance of the Sputnik 2 compared to the Sputnik 1 [42]. The main aim of current work was to evaluate the hemolysis level in flow path of the Sputnik LVADs using CFD prediction and experimental assessment as two different methods of evaluation. An additional aim was to evaluate other types of blood damage with CFD predictions.

## 2. Materials and Methods

### 2.1. Design Features

As both Sputnik LVADs are continuous-flow RBPs of axial type, main elements of their flow path are a fixed flow straightener, a fixed diffuser and a rotor represented by an impeller. All components are housed in a cylindrical flow tube. Straighteners are of similar design with three straight blades allocated at 120°. Impellers have two pairs of twisted blades placed on central hubs of different length and diameter. Due to this difference, the geometric shape of the diffusers also differs. The diffusers have three blades of different height twisted in the opposite direction to impeller blades and placed on different central hubs. Main design parameters of two generations of the Sputnik LVAD are represented in Table 1.

In comparison to the Sputnik 1, both impeller and diffuser in the Sputnik 2 were designed to exclude the impeller outlet taper unit, with an axial gap (0.5 mm) appearing between their central hubs. The distance between trailing edge of impeller blades and leading edge of diffuser blades was decreased from 20 mm to 3.6 mm. Diameter of the flow path was decreased from 16 mm to 14 mm along with a decreased clearance gap between impeller blades and flow path housing from 0.2 mm to 0.1 mm. Moreover, clearance gap between impeller central hub and diffuser blades was also decreased from 0.3 mm to 0.15 mm. Overall changes made possible the reduction of length and weight, and hence, the invasiveness of the implantable device.

**Figure 1:**
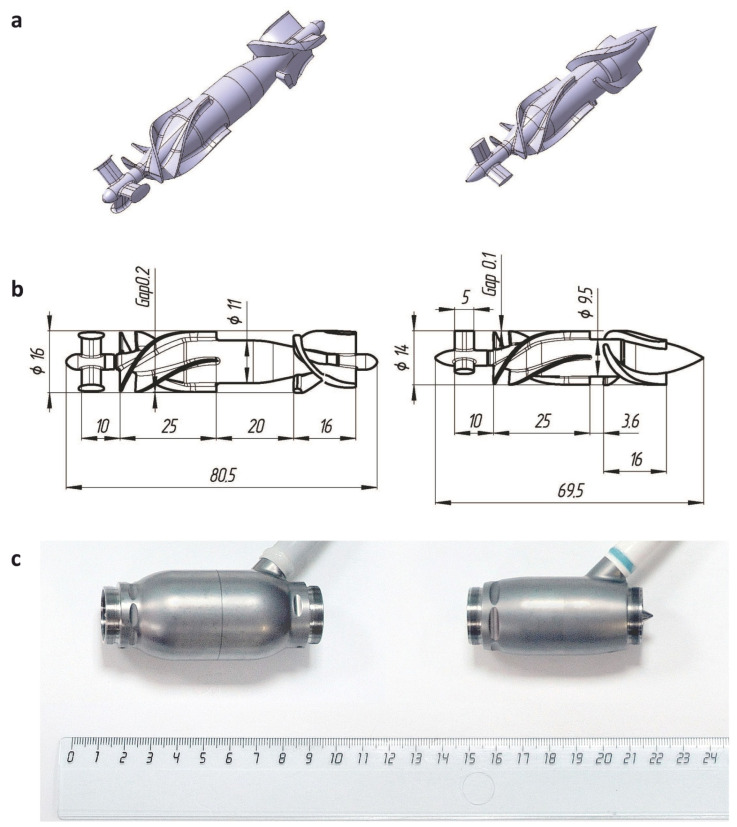
Design features of the Sputnik 1 (**left**) and Sputnik 2 (**right**) LVADs: (**a**) geometric models of main flow path elements (**left**-to-**right**: flow straightener, impeller, diffuser) utilized for investigation of pump influence on the blood damage level using methods of computational fluid dynamics; (**b**) frontal views of geometric models with applied dimensions; and (**c**) image of industrial samples with length scale, utilized for experimental assessment of hemolytic performance.

Investigation of pump influence on the level of blood damage with CFD was carried out for geometric models of the Sputnik 1 and Sputnik 2 LVADs, shown along with frontal views of models with applied dimensions and images of industrial samples in Figure 1. In industrial samples of the pumps, the main flow path elements are made of titanium alloy characterized by high durability-to-weight ratio and high compatibility to biological fluids and tissues. Cup-socket bearing pairs are connected to hub ends of corresponding elements at inlet (straightener and impeller) and outlet (impeller and diffuser) in both pumps. They are made of cobalt–chromium–molybdenum alloy additionally coated with diamond-like carbon (DLC). High thermal conductivity and hardness of this DLC-coated alloy are of primary importance, as it allows to substantially reduce heating and wear of bearing surfaces. However, most of the bearing surface is not washed with blood during operation in both designs giving an opportunity to exclude them from geometric models in CFD simulation.

### 2.2. CFD Simulations

Simulation of the flow in the pumps was carried out with commercial CFD software ANSYS FLUENT 19.0 (ANSYS Inc., Canonsburg, PA, USA) using pressure-based solver that takes into account the second order of discretization of parameter values.

The shear stress transport (SST) k-ω turbulence model was chosen to solve the three-dimensional Reynolds-averaged Navier–Stokes equations of the flow, because this model operates as the k-ω model in zones spaced from the walls and as k-ε model in the near-wall layers, thus, describing the flow behavior more precisely [10,11,21]. The discrete phase model (DPM) was used to calculate the trajectory of particles simulating red blood cells, allowing to determine flow impact on behavior of RBCs based on the Lagrangian approach.

Convergence criterion for numerical simulations (residual of continuity, velocity, turbulent kinetic energy, and specific dissipation rate) was set to 10^−4^ to provide sufficient accuracy and reasonable computational time.

#### 2.2.1. Meshing

Meshes for geometric models of both pumps were created with commercial grid generator software ANSYS MESHING. Meshes consist of unstructured hexahedrons with a characteristic size from 0.005 mm to 1 mm in different volumes of the computational domain. Appropriate resolution within the clearance gaps between impeller blades and flow path housing, and between impeller central hub and diffuser blades, in both Sputnik LVADs was implemented with at least 10 layers of cells, ensuring the convergence of iterative computational process.

In order to lower the computational effort, the number of elements in the outlet domain was reduced, since large gradients of the flow parameters were not likely to be observed in that region. The entire computational domain consisted of about 5.8 M and 4.4 M cells for Sputnik 1 and Sputnik 2, respectively.

#### 2.2.2. Hemolysis Estimation

As it was mentioned, shear stress and exposure time are the factors mostly considered by LVAD designers with regard to hemolysis. Giersiepen et al. [43] suggested a power law model for hemolysis estimation with CFD as shown in the Equation (1):

(1) $$HI\left(\%\right)=\frac{dHb}{Hb}\times 100=C\tau^{\alpha }T^{\beta },$$

where dHb/Hb is the dimensionless index of hemolysis (HI); τ is the scalar shear stress (Pa); T is the exposure time (s) to the corresponding stress; and C, α, and β are constants depending on the flow conditions.

In this study, $C=1.800\times 10^{-6}$, α=1.991 and β=0.765, since these values were shown to be the most appropriate for the Lagrangian approach of hemolysis estimation in axial RBPs [44,45]. Index of hemolysis quantifies red blood cells damaged by increased shear stress. In the Lagrangian formulation, index of hemolysis is integrated along the particle trajectories [46] or streamlines [7,8,22,23]. After solving constitutive equations of the flow, the trajectories are calculated from inlet to outlet of the computational domain. Then, trajectories are time-sampled, and damage to a particle p over a time step s appears to be calculated as follows:

(2) $$d_{p,s}=1.800\times 10^{-6}\times \tau_{p,s}^{1.991}\times \Delta t_{p,s}^{0.765},$$

where $\Delta t_{p,s}=t_{p,s}-t_{p,s-1}$ is the s-th time step for the p-th particle, with s=1,…,S and p=1,2,…,N. S is the index corresponding to final time instant for a particle leaving the pump, and N is a total number of particles in the simulation.

Damage to a particle p intensifies through the pump according to the Equation (3):

(3) $$D_{p,s}=D_{p,s-1}+\left(1-D_{p,s-1}\right)d_{p,s}.$$

Since in CFD simulations zero damage is assumed at the initial time instant, then $D_{p,\;0}=0$. The ultimate damage to a particle p after passing through the pump is $D_{p}=D_{p,S}$. Eventually, hemolysis index E of the pump is defined as a mean damage to all particles [7,10,31,46]:

(4) $$E=\sum_{p=1}^{N}\frac{D_{p}}{N}.$$

#### 2.2.3. Blood Damage Evaluation

Evaluation of blood damage can be performed based on the volumetric distribution of scalar shear stresses (SSS). The degree of shear stress influence can be characterized by threshold values of 9 Pa, 50 Pa and 150 Pa, as they are generally linked in literature to vWF degradation, platelet activation and RBCs destruction, respectively [12,14,15,28]. Calculation of scalar shear stress τ was carried out according to the Equation (5) based on viscous stresses by the methods described in the literature [21,47]. Viscous stresses are determined by the values of velocity gradient in the computational domain. The indices *i*, *j*, and *k* correspond to directions of the *x*, *y*, and *z* axes in a fixed Cartesian coordinate system:

(5) $$\tau ={\left[\frac{1}{3}\left(\tau_{ii}^{2}+\tau_{jj}^{2}+\tau_{kk}^{2}\right)-\frac{1}{3}\left(\tau_{ii}\tau_{jj}+\tau_{jj}\tau_{kk}+\tau_{kk}\tau_{ii}\right)+\left(\tau_{ij}^{2}+\tau_{jk}^{2}+\tau_{ki}^{2}\right)\right]}^{1/2},\;\tau_{ii}=-2\mu \frac{\partial V_{x}}{\partial x},\;\tau_{jj}=-2\mu \frac{\partial V_{y}}{\partial y},\;\tau_{kk}=-2\mu \frac{\partial V_{z}}{\partial z},\;\tau_{ij}=-\mu \left(\frac{\partial V_{x}}{\partial y}+\frac{\partial V_{y}}{\partial x}\right),\;\tau_{jk}=-\mu \left(\frac{\partial V_{y}}{\partial z}+\frac{\partial V_{z}}{\partial y}\right),\;\tau_{ki}=-\mu \left(\frac{\partial V_{z}}{\partial x}+\frac{\partial V_{x}}{\partial z}\right),$$

where μ is the dynamic fluid viscosity; and $V_{x}$, $V_{y}$, and $V_{z}$ are the components of the flow velocity vector.

#### 2.2.4. Boundary and Initial Conditions

The simulations were performed at the operating point corresponding to a flow rate of 5 L/min, pressure head of 100 mm Hg and impeller speed of 9100 min^−1^ for both pumps. This operating point was chosen to follow the required conditions for typical application of LVADs [48]. Fluid with constant viscosity of 3.5 mPa×s and density of 1055 kg/m^3^ was used as it is commonly utilized in CFD simulations of RBP operation [12,13,14,15,16,27,28,29,30,31,33,37].

For the simulations, 400 spherical particles with diameter of 7 μm and density of 1106 kg/m^3^ were used as basic RBCs. Mesh nodes at the inlet section of computational domain were the source of particles. The total mass flow of particles was 0.0392 kg/s with initial velocity of 1 mm/s along the normal to the inlet section. The value of time step along the particle trajectories had mean of 0.2 ms.

### 2.3. Experimental Set-Up

According to American Society of Testing and Materials (ASTM) standard [48], a series of experiments with industrial samples of the Sputnik 1 and Sputnik 2 LVADs were performed to assess the normalized index of hemolysis (NIH), which characterizes the degree of free hemoglobin in plasma (per 100 L of pumped blood), taking into account the blood hematocrit, blood flow rate and time of circulation. Three experiments were performed for Sputnik 1 and four experiments, for Sputnik 2. Each experiment was carried out on a separate day.

**Figure 2:**
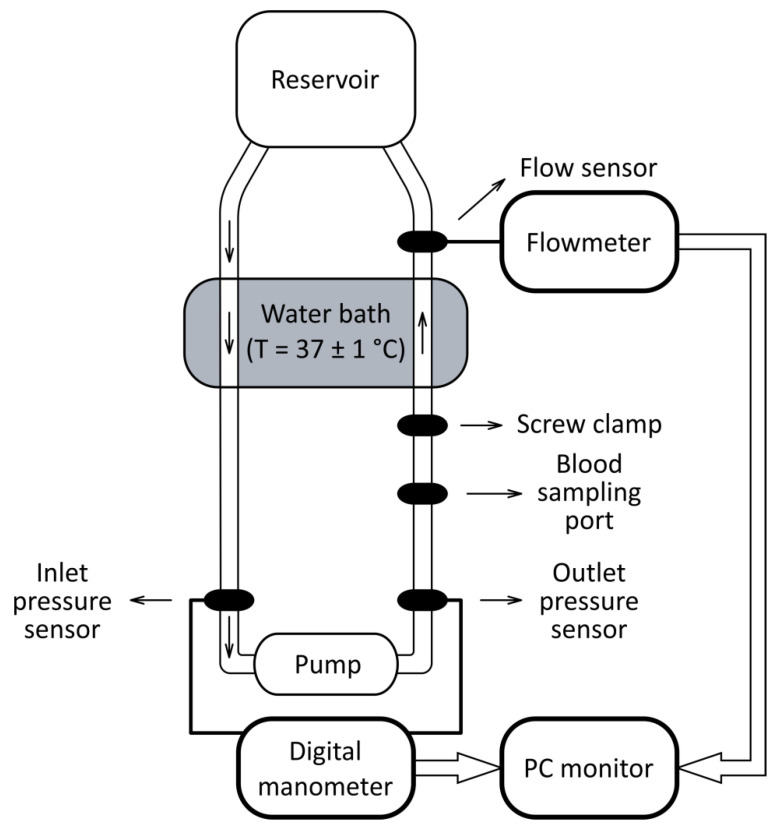
Functional diagram of the static circulation loop for experimental hemolysis assessment.

For the study, a static circulation loop (SCL), including a venous reservoir Capiox 400 mL (Terumo Corp., Tokyo, Japan) and an adjustable clamp allowing to change resistance of the loop, was used (Figure 2). All components of the SCL were connected with flexible polyvinyl chloride laboratory tubes (TYGON E-3603; Compagnie de Saint-Gobain, Courbevoie, Ile-de-France, France) and connectors. During experiments, the temperature of the blood in the SCL was maintained at 37 ± 1° C using a water bath with a thermostat. Tubes of the SCL were submerged into water allowing heat exchange and blood temperature maintenance.

An ultrasonic flow sensor ME11PXL349 and a flow meter module TS410 (Transonic Systems Inc., Ithaca, NY, USA) were used to measure blood flow rate in the SCL. Inlet and outlet pressure was measured with pressure sensors (TruWave; Edwards Lifesciences Corp., Irvine, CA, USA) connected to multichannel unit (ANGIOTON-4K; Biosoft-M, Moscow, Russia). Rotational speed of the impeller was set by means of a servo-controller for sensorless EC motors (ESCON Module 50/4 EC-S with ESCON Module Motherboard; maxon motor AG, Sachseln, Switzerland). Motherboard was connected to PC with ESCON Studio to adjust impeller speed.

Operating modes of the Sputnik 1 and Sputnik 2 LVADs were set according to requirements of ASTM standard [48]. In both pumps, impeller speed, pressure head and blood flow rate were set to 9100 min^−1^, 100 ± 10 mm Hg, and 5.0 ± 0.1 L/min, respectively.

#### 2.3.1. Donor Blood Preparation and the SCL Filling

Experiments with human blood were conducted in accordance with the Declaration of Helsinki, and the protocol was approved by the Ethics Committee of the Bakulev Scientific Center for Cardiovascular Surgery (Project identification code No 3 of 20 September 2016). In accordance with ASTM standards [48,49], human donor blood with hematocrit level of 30 ± 2% was used. To adjust hematocrit levels, dilution with 0.9% saline solution was performed. All donors were volunteers, gave informed consent and underwent standard screening procedures for normal body temperature, acceptable hematological profile ranges, and no signs of diseases, such as diarrhea and rhinorrhea. Additionally, donor blood was tested for no signs of HIV, viruses of hepatitis B and C, and syphilis.

Donor blood (500 ± 25 mL) was collected by vascular puncture and stored in standard bags with citrate phosphate dextrose adenine (CPDA-1) anticoagulant solution. Two bags of donor blood were provided for each experiment: one for filling of the SCL and one for control samples of self-hemolysis. SCL was filled with 450 ± 45 mL of donor blood within one hour after the collection. Air bubbles were carefully removed out of the SCL through input port of the venous reservoir.

#### 2.3.2. Blood Sampling and Hemolysis Assessment

The first blood sampling was made 5 min after the adjustment of LVADs operating mode, and then every 60 min. Overall duration of each experiment was 360 min from the first sampling. Thus, 14 samples (7 from the SCL and 7 control samples) were collected for hemolysis assessment.

**Figure 3:**
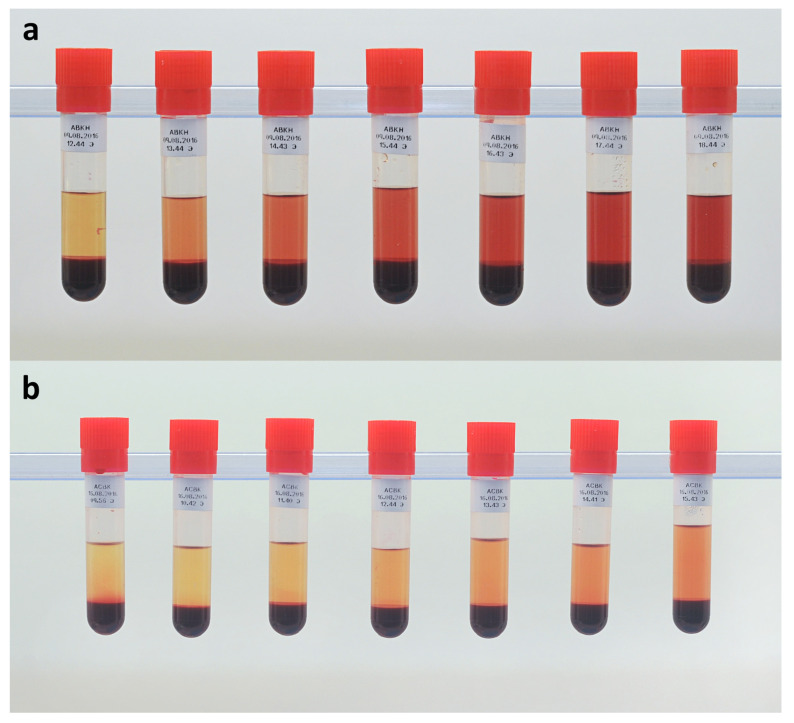
Example of blood samples in test-tubes after refrigeration obtained for hemolysis assessment: (**a**) Sputnik 1 LVAD; and (**b**) Sputnik 2 LVAD.

Before each sampling, a small amount of blood was drained out of the sampling port to avoid collection of stagnant blood. Thereafter, 2 mL of blood were sampled with a disposable syringe, poured into a test-tube marked with sampling time, and kept in a refrigerator at 4° C for 6 h to form a stable layer of plasma. Images of obtained blood samples after refrigeration are represented in Figure 3.

After precipitation of blood particles, supernatant plasma was transferred into quartz cells with a 1000 μL dispenser. The absorbance was measured with a spectrophotometer (Genesys 10S UV-Vis; Thermo Fisher Scientific, Waltham, MA, USA) in the specter of 300–700 nm in 0.2 nm step.

Concentration of plasma-free hemoglobin was determined by the absorbance at wavelengths of 560 nm, 576.4 nm and 592.8 nm [50]. NIH was calculated according to the following equation:

(6) $$\mathrm{NIH}\left(g/100\;L\right)=\Delta pfHb\times V\times \frac{100}{Q\times T}\times \frac{100-Ht}{100},$$

where ΔpfHb is an increase in concentration of plasma-free hemoglobin over the sampling time interval (g/L), *V* is the total blood volume in the SCL (L), *Ht* is the hematocrit level (%), *Q* is the blood flow rate (L/min), and *T* is the time interval of blood sampling (min).

## 3. Results

### 3.1. CFD Predictions

**Figure 4:**
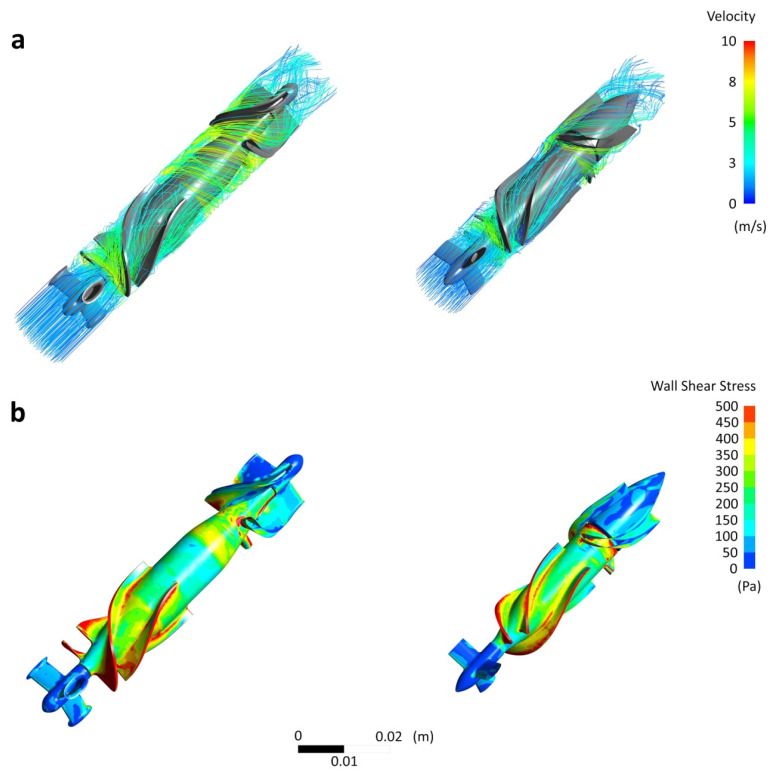
Visualization of the effects of flow path in the Sputnik 1 (**left**) and Sputnik 2 (**right**) LVADs at the operating point with flow rate of 5 L/min and pressure head of 100 mmHg at impeller speed of 9100 min^−1^: (**a**) streamlines colored by velocity; (**b**) wall shear stresses on the surfaces of flow straightener, impeller and diffuser.

Streamlines obtained for the Sputnik 1 and Sputnik 2 LVADs at the operating point with flow rate of 5 L/min and pressure head of 100 mmHg at impeller speed of 9100 min^−1^ are shown in Figure 4. The mean times of particle residence in flow path were 0.2159 s for the Sputnik 1 and 0.0394 s for the Sputnik 2. High velocities formed at impeller inlet and closer to transition region from impeller to diffuser in both pumps, because flow accelerated by the rotating part moving at several thousand rotations per minute passes towards the fixed part of the pump colliding with blades of the diffuser. Velocity in the region of impeller inlet was higher in the Sputnik 1. The maximum values of velocity were 9.14 m/s at the leading edge of impeller blades in the Sputnik 1 and 7.67 m/s at the leading edge of diffuser blades in the Sputnik 2.

As shown in Figure 4, walls of diffuser blades and most of the impeller surface in both pumps were the areas with values of wall shear stresses (WSS) as high or higher than 150 Pa. Total surface area with WSS higher than 150 Pa were 2413 mm² and 1586 mm² that are 29.7% and 27.5% of total surface area in flow path of the Sputnik 1 and the Sputnik 2, respectively. However, there were areas of substantially higher WSS in both pumps. For the Sputnik 1, WSS of 1300–3500 Pa were observed at the leading edge and along the tip edge of impeller blades. For the Sputnik 2, WSS of 600–1200 Pa were observed at the leading edge and along the tip edge of impeller blades, at the leading edge of diffuser blades and around the trailing edge of impeller hub. The calculated maximum WSS values were 5064.5 Pa and 1699.7 Pa for the Sputnik 1 and the Sputnik 2, respectively.

Potential thrombogenic surfaces with WSS below 5 Pa had similar total areas of 13.8 mm² and 13.7 mm² that are 0.17% and 0.23% of total surface area in flow path of the Sputnik 1 and the Sputnik 2, respectively.

**Figure 5:**
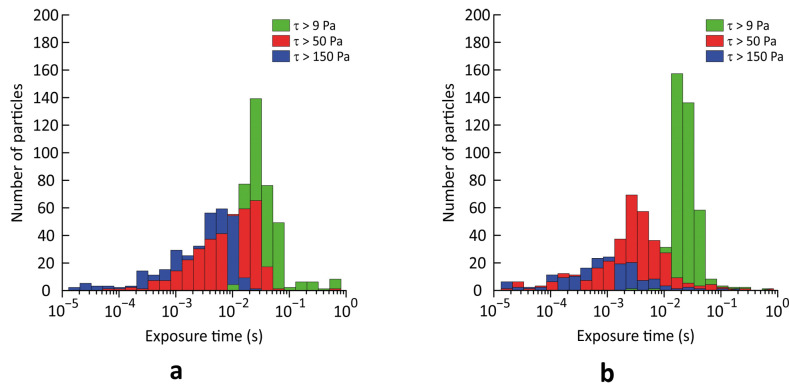
Bar charts for number of particles exposed to scalar shear stresses above 9 Pa (green), 50 Pa (red) and 150 Pa (blue): (**a**) Sputnik 1 LVAD; and (**b**) Sputnik 2 LVAD.

Bar charts for number of particles with respect to the time of exposure to SSS above threshold values of 9 Pa, 50 Pa and 150 Pa are shown in Figure 5. In the Sputnik 1, 85%, 83% and 79% of particles experienced SSS above 9 Pa, 50 Pa and 150 Pa, respectively, compared to 92%, 88% and 41% of particles in the Sputnik 2. Hence, total number of particles exposed to SSS above 150 Pa was almost two times greater and exposure times for most of these particles were longer in the Sputnik 1 compared to the Sputnik 2. For particles exposed to SSS above 50 Pa, exposure times were longer in the Sputnik 1, as well. In contrast, exposure times to SSS above 9 Pa were slightly longer in the Sputnik 2 compared to the Sputnik 1.

The maximum values of volumetric SSS calculated for both pumps were the same as for WSS. These values were observed in the region of clearance gap between impeller blades and flow path housing in the Sputnik 1, and in the region of gap between impeller central hub and diffuser blades in the Sputnik 2. However, most of volume in those regions was exposed to SSS in the ranges of 1300–3500 Pa and 1100–1400 Pa for the Sputnik 1 and the Sputnik 2, respectively.

The calculated mean hemolysis indices E of Lagrangian approach are 0.0090% and 0.0023% for the Sputnik 1 and the Sputnik 2, respectively, at the operating point. Thus, this index is more than 3.5 times lower in the Sputnik 2 compared to the Sputnik 1.

### 3.2. Experimental Hemolysis Assessment

**Figure 6:**
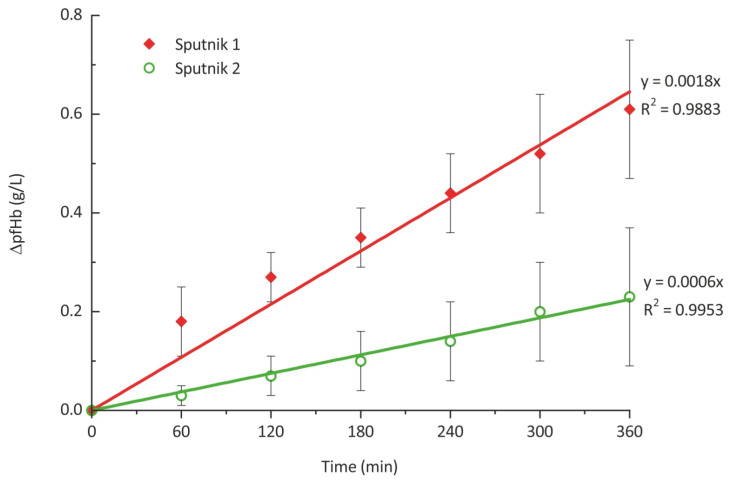
Change in concentration of plasma-free hemoglobin with error bars, averaged over 3 and 4 experiments for Sputnik 1 and Sputnik 2 LVADs, respectively.

Changes in concentration of plasma-free hemoglobin averaged for three and four experiments performed for the Sputnik 1 and Sputnik 2, respectively, are represented in Figure 6. According to ASTM standard [48], linear regression curves with a coefficient of determination above 0.95 were obtained. As one can see, Sputnik 2 demonstrates less change in concentration of plasma-free hemoglobin even given the margin of error. Although the overall trend of concentration change is linear for the Sputnik 1, it can be seen that in average of experiments, concentration was changing more significantly during the first hour than during any other hour of experiments. For Sputnik 2, free hemoglobin concentration was changing more gradually and evenly over entire experiment duration. Concentration of plasma-free hemoglobin for the Sputnik 1 and Sputnik 2 after 6 h of circulation was 0.61 ± 0.14 g/L and 0.23 ± 0.14 g/L, respectively.

As another result, NIH values of 0.011 ± 0.003 g/100 L and 0.004 ± 0.002 g/100 L were obtained for the Sputnik 1 and Sputnik 2, respectively. The critical value of NIH for LVADs is 0.01 g/100 L [51]. Thus, given the margin of error, both Sputnik 1 and Sputnik 2 can be considered having acceptable impact on RBCs. However, Sputnik 1 is too close to the critical value, implying the need of design improvements. Additionally, NIH for the Sputnik 2 compared to the Sputnik 1 was decreased more than 2.5 times, indicating that represented design changes allow to substantially decrease impact on RBCs.

## 4. Discussion

Flow fields and potential differences in hemolytic performance of the Sputnik 1 and Sputnik 2 LVADs were in focus of the present study. Geometric parameters of flow path in the pumps were compared with CFD predictions and experimental assessment of hemolysis. Impact on RBCs was examined in terms of indices of hemolysis commonly used in CFD and experiments. However, it is impossible to determine a particular part of the design, where degree of damage to RBCs reaches its maximum value, using only the numerical indices. Furthermore, hemolysis is not the only type of blood damage occurring in the flow path of RBPs. Therefore, in addition to hemolysis indices, flow path geometry was evaluated with exposure times and distribution of shear stresses concerning established thresholds for different types of blood damage. These thresholds corresponding to vWF degradation (9 Pa), platelet activation (50 Pa) and hemolysis (150 Pa) were taken from the literature [12,14,15,28]. Since other values for different types of blood damage were also reported [11,27,29,39], it should be noted that choice of threshold levels may affect the results. Although a particular threshold value does not take into account the actual damaging effect, this classification method is still simple and expressive enough to characterize and compare devices. Besides, it is commonly employed by different research groups in combination with other indices of blood damage.

During last two decades, implications on predictions of blood damage of various changes in different aspects of LVAD design were substantially described with CFD simulations. RBP designers mainly face two requirements at this course: highly efficient hydraulic performance and minimization of the impact on blood cells for improved hemocompatibility. Geometric parameters and number of impeller blades [28,32,33,34,35,36,37,39], size of clearance gap between impeller blades and flow path housing [28,29,38], distance between trailing edge of impeller blades and leading edge of diffuser blades, clearance gap between diffuser blades and impeller central hub [27,29,31], and number of diffuser blades [30,31] were shown to be design parameters influencing the hydraulic efficiency and the predictions of blood damage. These parameters were studied for different basic designs of both axial and centrifugal RBPs with the only one parameter varied and others fixed. Although in the present study overall design changes of flow path in the Sputnik LVADs can only be considered and evaluated as a whole, and impact of no one parameter changed should be evaluated separately from the other changes, it would be better to see into how those separate changes can influence the blood damage to suppose a net effect and relate it to the obtained results.

In the literature, among geometric parameters affecting hydraulic efficiency and blood damage, both mentioned clearance gaps were shown to have the strongest influence on the level of maximum volumetric scalar shear stress in the flow path. Although size of the gap inversely related with the maximum SSS, larger gaps induce significant flow disturbance, vortices and leakage flow, increasing the regions of high shear stress or the exposure time. Thus, smaller gaps were shown to have less hemolytic impact in axial [27,29] and centrifugal RBPs [28,38], though causing increase of stagnation regions with low particle velocity [28,29]. In combination with higher hydraulic efficiency this makes smaller gaps more eligible in design [28,29].

In both Sputnik LVADs represented, both considered clearance gaps between impeller blades and flow path housing, and between diffuser blades and impeller hub, are small. Although maximum values of volumetric scalar shear stress in the region of these gaps are very high in both designs, particular results indicate substantial improvement of these values in the Sputnik 2 compared to the Sputnik 1. Beside almost threefold decrease in the maximum level, the range of volumetric SSS values in most volume of the gap regions was narrowed down more than seven times. Exposure time to volumetric SSS above 150 Pa was decreased by an order in the Sputnik 2 for most of particles. Furthermore, the number of particles exposed to SSS of or above 150 Pa decreased by two times in the Sputnik 2.

Despite wall shear stresses of 150 Pa or higher occur almost throughout the impeller surface in both Sputnik LVADs, total surface area with these WSS decreased more than 1.5 times in the Sputnik 2. This was achieved due to decrease in diameter of impeller and the distance between trailing edge of impeller blades and leading edge of diffuser blades. Additionally, these changes provided the decrease in the maximum level of flow velocity and more than fivefold decrease in the mean-time of particle residence in flow path, reducing the risk of exposure to high shear stresses.

Mean hemolysis index E for the Sputnik 2 is more than 3.5 times less than for the Sputnik 1. NIH is also more than 2.5 times less for the Sputnik 2. Although direct comparison of these indices is rather improper, comparison of relative changes indicates alike tendency of improvement in hemolytic performance of the Sputnik 2, which also confirms other CFD predictions. NIH of clinically used HeartMate II (Abbott Laboratories, Abbott Park, IL, USA) and HeartWare HVAD (HVAD; Medtronic, Minneapolis, MN, USA) reported for similar experiments with human blood was 0.00525 ± 0.00183 g/100 L and 0.00583 ± 0.00182 g/100 L, respectively [52]. Obviously, NIH value for the Sputnik 1 is about 2 times larger but NIH value for the Sputnik 2 is lesser than these values. However, such comparison of results may also be improper due to some differences in experimental setup and investigated operating point. The same experimental procedure was performed for HeartMate II (HMII) and HeartMate 3 (HM3; Abbott Laboratories, Abbott Park, IL, USA) at the same operating point (i.e., flow rate of 5 L/min at 100 mm Hg) with bovine blood [53]. Concentration of plasma-free hemoglobin at 6 h of circulation was reported as 1.23 g/L and 0.74 g/L for HMII and HM3, respectively. These values are larger than those obtained in both Sputnik LVADs.

Although a similar number of particles was exposed to volumetric SSS above 9 Pa and 50 Pa in both designs, exposure times to SSS above 50 Pa were shorter in the Sputnik 2, implying a decrease of impact on platelet activation. However, exposure times to SSS above 9 Pa were slightly longer for most of particles in the Sputnik 2, implying no considerable change of impact on vWF degradation. Moreover, total surface areas of potential thrombus formation have similar values in both represented designs, which, however, are lesser than reported thrombogenic surface areas in clinically utilized HMII and HVAD [15].

Overall obtained results of CFD predictions and experimental assessment of impact on the blood indicate obvious improvements in hemolytic performance for the Sputnik 2 compared to the Sputnik 1. Results also suggest that Sputnik 2 may have better hemolytic performance than LVADs widely utilized in clinics around the world. However, this matter should be confirmed in a separate study with these devices included as subjects. Additionally, implantable blood pumps of both Sputnik LVADs have weight and size parameters comparable to HMII, HM3 and HVAD systems. Weight of these blood pumps is 281 g, 200 g and 160 g, respectively [52]. Dimensions of HMII pump body are 43 mm of diameter and 81 mm of length. HM3 blood pump has a diameter of 50.3 mm and height of 33.8 mm [53]. The diameter of the pump body is 34 mm and 29 mm and the length is 82 mm and 70.5 mm for the Sputnik 1 and Sputnik 2, respectively. Thus, the miniaturization aim in the Sputnik 2 development was sufficiently satisfied implying required decrease of invasiveness along with improvement in hemocompatibility and versatility for patients of various sizes.

Translational and clinical challenges for implementation of the Sputnik 2 would be the same as for the Sputnik 1 that was successfully introduced into clinical practice. Design of the Sputnik 2 provides proper connection to the cardiovascular system in the same way and with the same inflow and outflow elements utilized for the Sputnik 1. Therefore, implantation procedure would not change considerably. Additionally, use of bio- and hemocompatible materials such as titanium alloy, cobalt–chromium–molybdenum alloy and DLC ensures suitability for clinical use [54]. Furthermore, in the case of Sputnik 1, manufacturing of a diffuser is the most technologically difficult process due to complex geometry, which leads to high risk of defects, and hence, raises the overall cost of LVAD. In the Sputnik 2, geometry of diffuser was intentionally simplified to facilitate improved manufacturing quality and reduced costs.

The study findings have to be seen in light of some limitations. First, non-Newtonian blood characteristics were not considered. The viscosity becomes independent of the shear stress at shear rates above 100 s^−1^ [14,15,16], which suggests that blood can be considered as Newtonian fluid. Although many models consider non-Newtonian rheological properties of blood, there is no standardized blood model [55,56,57]. Therefore, assumption of constant viscosity of whole blood is commonly utilized [58,59,60,61,62,63]. In most blood volumes of both Sputnik pumps, the shear rates are higher than 100 s^−1^. These facts allowed us to use the Newtonian blood flow model (constant viscosity of whole blood). However, shear thinning property can affect pump performance. In particular, the pressure increase at low flow rates is less for shear thinning fluid than for Newtonian fluid [64]. Hence, better accuracy of CFD predictions in LVADs may be achieved with non-Newtonian blood models [65,66].

Second, RBCs were repeatedly exposed to high shear stresses in the experiments, but only once in the CFD simulations. Although hemolysis generally does not occur at shear stresses below 150 Pa [25,26], it is not known whether RBCs are destroyed being repeatedly exposed to these shear stresses. It is also not known whether the non-lethally damaged cell membrane restores with time. Thus, a direct comparison between results of experiments and CFD simulations seems to be inappropriate and should not be performed.

Third, even though wall shear stress analysis was performed considering the axial gap between hubs of impeller and diffuser in the Sputnik 2, issue of particles passed through or caught in this region was not investigated. Despite experiments did not reveal any thrombus formation inside both Sputnik LVADs, their character and duration may be insufficient for this purpose. This gap appears to be potentially thrombogenic region and should be studied more thoroughly. Furthermore, experimental assessment of impact on vWF degradation should be performed to validate the obtained results of CFD prediction.

Finally, simulations and experiments were performed in conditions of continuous flow with constant flow rate and pressure head. Evaluation of pulsatile-flow conditions should be performed to consider the influence of physiological pulsations on blood damage in the cardiovascular system and the inherent functions of LVAD, such as washout algorithms.

## 5. Conclusions

Presented results of both numerical prediction and experimental assessment of hemolytic performance in the Sputnik LVADs revealed considerable improvements in the Sputnik 2. Moreover, numerically predicted results for other types of blood damage also indicate potential advances of the Sputnik 2 compared to the Sputnik 1. In summary, improved hemocompatibility and decreased invasiveness of the Sputnik 2, along with reduced power consumption and increased hydraulic performance shown in previous studies, confirm successful attainment of miniaturization aim in the entire process of design and development.

## Figures and Tables

**Table 1:** Difference in parameters of the Sputnik 1 and Sputnik 2 LVADs.

LVAD Generation	Flow Path Length, mm	Impeller Outer Diameter, mm	Clearance Gap, mm		Distance between Trailing Edge of Impeller Blades and Leading Edge of Diffuser Blades, mm	Overall Pump Weight, g
			Between Impeller Blades and Housing	Between Impeller Hub and Diffuser Blades		
Sputnik 1	80.5	15.6	0.2	0.3	20.0	246
Sputnik 2	69.5	13.8	0.1	0.15	3.6	205

## Data Availability

The datasets obtained and analyzed during the current study are available from the corresponding authors on reasonable request.
